# Draft genome of the Chinese mitten crab, *Eriocheir sinensis*

**DOI:** 10.1186/s13742-016-0112-y

**Published:** 2016-01-28

**Authors:** Linsheng Song, Chao Bian, Yongju Luo, Lingling Wang, Xinxin You, Jia Li, Ying Qiu, Xingyu Ma, Zhifei Zhu, Liang Ma, Zhaogen Wang, Ying Lei, Jun Qiang, Hongxia Li, Juhua Yu, Alex Wong, Junmin Xu, Qiong Shi, Pao Xu

**Affiliations:** Key Laboratory of Freshwater Fisheries and Germplasm Resources Utilization, Ministry of Agriculture, Freshwater Fisheries Research Center, Chinese Academy of Fishery Sciences, Wuxi, 214081 China; College of Fisheries and Life Science, Dalian Ocean University, Dalian, 116023 China; Shenzhen Key Lab of Marine Genomics, Guangdong Provincial Key Lab of Molecular Breeding in Marine Economic Animals, BGI, Shenzhen, 518083 China; Guangxi Academy of Fisher Sciences, Nanning, 530021 China; Key Laboratory of Experimental Marine Biology, Institute of Oceanology, Chinese Academy of Sciences, Qingdao, 266071 China; BGI Zhenjiang Institute of Hydrobiology, Zhenjiang, 212000 China; Zhenjiang Agriculture Committee, Zhenjiang, 212000 China; BGI-Hong Kong, Hong Kong, 999077 China

**Keywords:** Crab genome, Genomics, Assembly, Annotation

## Abstract

**Background:**

The Chinese mitten crab, *Eriocheir sinensis*, is one of the most studied and economically important crustaceans in China. Its transition from a swimming to a crawling method of movement during early development, anadromous migration during growth, and catadromous migration during breeding have been attractive features for research. However, knowledge of the underlying molecular mechanisms that regulate these processes is still very limited.

**Findings:**

A total of 258.8 gigabases (Gb) of raw reads from whole-genome sequencing of the crab were generated by the Illumina HiSeq2000 platform. The final genome assembly (1.12 Gb), about 67.5 % of the estimated genome size (1.66 Gb), is composed of 17,553 scaffolds (>2 kb) with an N50 of 224 kb. We identified 14,436 genes using AUGUSTUS, of which 7,549 were shown to have significant supporting evidence using the GLEAN pipeline. This gene number is much greater than that of the horseshoe crab, and the annotation completeness, as evaluated by CEGMA, reached 66.9 %.

**Conclusions:**

We report the first genome sequencing, assembly, and annotation of the Chinese mitten crab. The assembled draft genome will provide a valuable resource for the study of essential developmental processes and genetic determination of important traits of the Chinese mitten crab, and also for investigating crustacean evolution.

## Data description

Genomic DNA was extracted from muscle tissue of a single female crab (*Eriocheir sinensis*; NCBI Taxonomy ID: 95602) after 3 generations of inbreeding that was obtained from a local farm in Panjin, Liaoning Province, China. We used the whole-genome shotgun sequencing strategy and constructed the subsequent short-insert libraries (170, 250, 500 and 800 bp) and long-insert libraries (2, 5, and 10 kb) using the standard protocol provided by Illumina (San Diego, USA). Paired-end sequencing was performed by the Illumina HiSeq 2000 system. In total, we generated 258.8 Gb of raw reads from all constructed libraries.

We extracted clean reads of the short-insert libraries (500 or 800 bp) to estimate the crab genome size by k-mer frequency distribution analysis [1]. A k-mer is related to an artificial sequence division of K nucleotides iteratively from sequencing reads. We defined the k-mer length as 17 bp; thus, a L bp-long clean sequence would include (L-17 + 1) k-mers. The frequency of each k-mer can be calculated from the genome sequence reads. Typically, k-mer frequencies were plotted against the sequence depth gradient following a Poisson distribution in any given dataset. The genome size (G), can be deduced from the formula:

$$ \mathrm{G} = \mathrm{N} \times \left(\mathrm{L}\hbox{-} 17+1\right)/\mathrm{K}\_\mathrm{depth} $$

where N is the total number of reads, and K_depth indicates the frequency that occurrs more often than other frequencies. In our calculations, N was 789,326,187 and K_depth was 40; therefore, the crab genome size was estimated to be 1.66 Gb.

For whole-genome assembly, we employed Platanus [2] with optimized parameters (−k 27, −m 200) to construct contigs and original scaffolds. All reads were mapped onto contigs for scaffold building by utilizing the paired-end information. This paired-end information was subsequently applied to link contigs into scaffolds using a stepwise approach. Some intra-scaffold gaps were filled by local software using read-pairs in which one end uniquely mapped to a contig and the other end was located within a gap. Final genome assembly of the Chinese mitten crab is 1.12 Gb in total length, which is about 67.5 % of the estimated genome size. The contig N50 size (i.e., 50 % of the genome is in fragments of this length or longer) is 6.02 kb, and the scaffold (>2 kb) N50 is 224 kb.

We constructed a *de novo* repeat library using RepeatModeller (Version 1.04, default parameter) and LTR_FINDER [3]. To identify known and *de novo* transposable elements (TEs), we employed RepeatMasker (Version 3.2.9) [4] against the Repbase TE library [5] (Version 14.04) and the *de novo* repeat library. In addition, we used RepeatProteinMask (Version 3.2.2) implemented in RepeatMasker to detect the TE-relevant proteins. We also predicted tandem repeats utilizing Tandem Repeat Finder [6, 7] (Version 4.04) with parameters set as “Match = 2, Mismatch = 7, Delta = 7, PM = 80, PI = 10, Minscore = 50, and MaxPerid = 2000”. Finally, we confirmed that the repeat sequences occupy approximately 50.4 % of the crab genome. Among them, the long interspersed elements, occupying 19.0 % of the crab genome, are the most predominant type of repeat sequences.

Subsequently, we performed annotation analysis containing four major steps. (1) The homology-based gene prediction: We aligned *Homo sapiens*, *Crassostrea gigas*, *Caenorhabditis elegans*, *Drosophila melanogaster* and *Daphnia pulex* proteins (Ensembl release 75) to the crab genome using TblastN with an E-value ≤ 1E-5, and then made use of GeneWise2.2.0 [7] for precise spliced alignment and predicting gene structures. Short genes (<150 bp) and premature or frame-shifted genes were removed. (2) The *ab initio* prediction: Genome sequences of the crab were repeat-masked, and 1500 full-length, randomly selected genes from their homology gene sets were used to train the model parameters for AUGUSTUS2.5 [8]. We then utilized AUGUSTUS2.5 and GENSCAN1.0 [9] for *de novo* prediction on repeat-masked genome sequences. Short genes were discarded using the same filter threshold that was used for homology prediction. (3) Gene structure identification using transcriptome reads: We mapped the mixed RNA reads (from hepatopancreas tissue taken from four molting stages) reported in Huang’s study [10] on the crab genome using TopHat1.2 [11]. Subsequently, we sorted and merged the TopHat mapping results and then applied Cufflink [12] software to identify gene structures to assist gene annotation. (4) Gene set integration: All of the above gene sets were merged to form a comprehensive and non-redundant gene set using GLEAN [13]. We obtained a final gene set containing 7,549 genes (Table 1), which is more than the gene number (5,775) identified for horseshoe crab [14]. Meanwhile, the CEGMA [15] evaluation demonstrated the annotation completeness to be 66.9 % (166 of 248 core eukaryote genes were aligned).

**Table 1:** Summary of genome annotations

		Number	Average transcript length (bp)	Average coding sequence length (bp)	Average exons per gene	Average exon length (bp)	Average intron length (bp)
*De novo*	AUGUSTUS	14,436	10,104	1,195	4.97	240	2,245
	Genescan	29,097	13,045	1,022	5.01	203	2,995
Homolog	*H. sapiens*	5,646	4,752	922	3.74	246	1,398
	*C. gigas*	9,470	3,067	641	2.69	238	1,432
	*C. elegans*	3,142	3,913	819	3.27	250	1,361
	*D.melanogaster*	4,369	6,178	981	4.31	227	1,571
*D. pulex*		14,183	2,887	628	2.48	252	1,521
Transcriptome		14,123	11,161	2,223	6.83	325	1,532
GLEAN		7,549	12,742	1,470	6.36	230	2,101

In summary, we report the first genome sequencing, assembly, and annotation of the Chinese mitten crab. The draft genome will provide a valuable resource for studying essential developmental processes in the Chinese mitten crab, investigating crustacean evolution, and improving the molecular breeding of this economically important species.

## Availability of supporting data

Supporting data are available in the GigaDB database [16], and the raw data were deposited in the PRJNA305216.

## Abbreviations

Gb: Gigabase
TE: Transposable element
